# The Planning of Maternity Hospitals and Homes

**Published:** 1920-03-20

**Authors:** 


### THE PLANNING OF MATERNITY HOSPITALS AND HOMES.

### Official Notes on Design and Organisation.

A MEMORANDUM which has just been issued by the Ministry of Health on Maternity Hospitals and Homes*, may be expected to exercise a strong influence over the future design and construction of this type of institution, more especially in country districts and towns not large enough to support the very large clinics such as London, Dublin, Edinburgh, Manchester can and do support.

The memorandum begins with certain general considerations on the need for, and on the distinctions which can be drawn between, these two types —the hospital and the maternity home. The former contemplates both larger numbers and more complicated cases than the latter, and also the possibility of being a teaching centre. It needs, therefore, to be more extensive and more fully equipped. It should have accommodation for a resident medical officer; and so on.

As regards site, it is officially laid down as desirable (evidently small towns are held in view, for the provision is obviously impossible in large towns) that an institution of twelve to eighteen beds should have a site of not less than an acre and a-half, and one for twenty to thirty beds of not less than two acres. It is considered that on sites of this size, flowers, fruit, and vegetables for patients and staff can be grown, but these paragraphs on sites are rather too vague, and even in places nonsensical to be of much help to committees: official publications should not encourage such slipshod and non-committal "hot-air." This is not the kind of thing that Sir Robert Morant was accustomed to tolerate in any branch of his administration.

### DESIGN AND PLANS.

Up to a limit of 24 beds a single building is favoured; above that an arrangement on the pavilion system is recommended. In the former type the patients are to occupy the ground, and, if necessary, also the first floors; and the staff quarters are to be above these. In the latter type a separate building for nurses and staff quarters is advised. It is not laid down, though it is perhaps to be inferred from the words used, that each nurse is to have a separate bedroom; this might well have received more emphasis. Neither washstands and basins nor fireplaces are advised for these bedrooms; the former are to be replaced by "sufficient lavatory accommodation on the first floor"; and the latter by radiators in the passages. Provided lavatory accommodation is understood to include sufficient hot and cold baths for the staff, there is something in this argument: the matron, by the way, is to have "perhaps a bathroom" of her own.

One nurse's bedroom is to have a fireplace of the ordinary kind for use in cases of sickness among the staff. For heating of the premises in general, low-pressure radiators are strongly pressed for in preference to open fires. A separate boiler, arranged to act also as a destructor, is recommended for the hot-water supply in the wards, bathrooms, etc. The sections on walls, floors, linen stores, and similar matters of hospital construction contains information of much practical value.

### ORGANISATION.

Maternity hospitals, it is contemplated, of more than twenty-five beds should have a resident medical officer and a visiting staff as well; one at least of these doctors to be a woman. Maternity homes require only that two or more medical practitioners having special experience of midwifery shall be available when required. The matron and sisters, the memorandum states, should have general training as well as training in midwifery. The minimum nursing staff is such as will provide on the average of one nurse for every three mothers and their babies by day and for every eight mothers and nurses by night.

### THE LINK WITH WELFARE CENTRES.

Paragraphs on septic cases, venereal cases, tuberculous patients, period of stay in the institution, pre-maternity accommodation, and "home-helps" lead on to the connection of maternity hospitals with infant welfare centres and children's hostels. The greater part of the memorandum, however, is taken by model plans of institutions of varying sizes and purposes, signed by the architects' department of the Ministry of Health. The most ambitious of these is for a maternity and child welfare hospital of 120 beds, arranged in four thirty-bed pavilions, with separate isolation administration, staff, and laundry blocks. But much smaller institutions, even down to six beds, are also shown.

Estimates of cost are not given; it is hardly possible that in the present state of the building trade they should be. Nevertheless, those who promote the erection of such homes and hospitals have to cut their coats according to their cloth; and it will seldom be possible to obtain at a feasible cost the highly advantageous types of buildings, space, and aspect here presented. As an ideal, to be approached as nearly as circumstances will permit, these model plans will save a useful purpose, however, and should prove of great value to health committees and other bodies proposing to incur responsibilities of this particular kind. Estimates of cost per occupied bed per annum or per patient received are not considered; but it is evident that local authorities will require to take all this very seriously into consideration.

* H.M. Stationery Office. Price 9d. net.

